# Two Cases of Actinomycosis Treated with Iodcalium

**Published:** 1896-06

**Authors:** J. Meisinger


Two Cases of Actinomycosis Treated with Iodcalium. By J. Meisinger. In August, 1894, I was called to treat a cow in Radstadt, which, on account of swelling of the tongue, could not eat, according to the statement of the owner. She stood slavering in the stall, her tongue hung about 2 cm. out of her mouth, was very considerably enlarged, and in places lumpy and hard. It could be moved only a little, and was yellowish in color. The cow had been nourished for about three weeks by pouring into her from a bottle a liquld mixture of bran and lentils. The owner did not call in a veterinary surgeon until she refused to take this mixture longer, after which time several veterinarians tried their skill in treating her for two or three months without success. I gave her 6 g. iodide of potassium dissolved in lukewarm water three days in succession. On August 16th she had drawn her tongue back somewhat, and a strong mattery-like excretion at the nose was present. The same treatment continued for three days longer attained the result that the cow began to eat for herself. When I examined the animal not long ago I found the tongue smaller and softer; excretion at the nose was no longer present. The same solution of potassium iodide was given continuously until Aug. 31st, when I declared the cow wholly recovered. She was sold in the year 1895 without anything unusual being noticed about her.

On the 2d of July, 1895, I was called to Ennswald, where I found a young cow with similar symptoms. The tongue was very much enlarged, hard as a board, immovable, and of a salamander-yellow. The swelling of the tongue in the throat-entrance could be felt as a hard spherical lump. The animal was likewise kept alive for weeks with liquid nourishment. I gave the same dose, as in the first case, for six days, and had the hard lump at the entrance of the throat painted with tincture of iodine. On the 11th of July the swelling in the throat was very small; the tongue was already softer and smaller. On September 20th she could be driven out to pasture, and began to eat slowly. The same treatment, without the tincture of iodine, was continued until the 28th of September, when the cow was considered cured.

A microscopical investigation was not possible; but, nevertheless, I believe, without doubt, that both were cases of actinomycosis, as the disease is frequently observed here, and swelling of the jaws occurs quite often.—*Ibid.*

